# Identification of a Potential Vaccine against *Treponema pallidum* Using Subtractive Proteomics and Reverse-Vaccinology Approaches

**DOI:** 10.3390/vaccines11010072

**Published:** 2022-12-28

**Authors:** Siyab Khan, Muhammad Rizwan, Adnan Zeb, Muhammad Alaa Eldeen, Said Hassan, Ashfaq Ur Rehman, Refaat A. Eid, Mohamed Samir A. Zaki, Ghadeer M. Albadrani, Ahmed E. Altyar, Nehal Ahmed Talaat Nouh, Mohamed M. Abdel-Daim, Amin Ullah

**Affiliations:** 1 School of Life Sciences, Northeast Normal University, Changchun 130024, China; 2 Center for Biotechnology and Microbiology, University of Swat, Kanju Campus, Swat 19120, Pakistan; 3 Department of Biotechnology, Quaid-i-Azam University, Islamabad 45320, Pakistan; 4 Cell Biology, Histology & Genetics Division, Biology Department, Faculty of Science, Zagazig University, Zagazig 44519, Egypt; 5 Institute of Biotechnology and Microbiology, Bacha Khan University Charsadda, Peshawar 24540, Pakistan; 6 Departments of Molecular Biology and Biochemistry, University of California, Irvine, CA 92697-3900, USA; 7 Department of Pathology, College of Medicine, King Khalid University, Abha 62529, Saudi Arabia; 8 Anatomy Department, College of Medicine, King Khalid University, Abha 62529, Saudi Arabia; 9 Department of Histology and Cell Biology, College of Medicine, Zagazig University, Zagazig 31527, Egypt; 10 Department of Biology, College of Science, Princess Nourah bint Abdulrahman University, Riyadh 11671, Saudi Arabia; 11 Department of Pharmacy Practice, Faculty of Pharmacy, King Abdulaziz University, Jeddah 21589, Saudi Arabia; 12 Department of Microbiology, Medicine Program, Batterjee Medical College, Jeddah 21442, Saudi Arabia; 13 Inpatient Pharmacy, Mansoura University Hospitals, Mansoura 35516, Egypt; 14 Department of Pharmaceutical Sciences, Pharmacy Program, Batterjee Medical College, Jeddah 21442, Saudi Arabia; 15 Pharmacology Department, Faculty of Veterinary Medicine, Suez Canal University, Ismailia 41522, Egypt; 16 Department of Health and Biological Sciences, Abasyn University Peshawar, Peshawar 25000, Pakistan

**Keywords:** *Treponema pallidum*, syphilis, immunoinformatic, epitopes, vaccine, subtractive proteomics, immune simulation, in silico cloning

## Abstract

Syphilis, a sexually transmitted infection, is a deadly disease caused by *Treponema pallidum*. It is a Gram-negative spirochete that can infect nearly every organ of the human body. It can be transmitted both sexually and perinatally. Since syphilis is the second most fatal sexually transmitted disease after AIDS, an efficient vaccine candidate is needed to establish long-term protection against infections by *T. pallidum.* This study used reverse-vaccinology-based immunoinformatic pathway subtractive proteomics to find the best antigenic proteins for multi-epitope vaccine production. Six essential virulent and antigenic proteins were identified, including the membrane lipoprotein TpN32 (UniProt ID: O07950), DNA translocase FtsK (UniProt ID: O83964), Protein Soj homolog (UniProt ID: O83296), site-determining protein (UniProt ID: F7IVD2), ABC transporter, ATP-binding protein (UniProt ID: O83930), and Sugar ABC superfamily ATP-binding cassette transporter, ABC protein (UniProt ID: O83782). We found that the multiepitope subunit vaccine consisting of 4 CTL, 4 HTL, and 11 B-cell epitopes mixed with the adjuvant TLR-2 agonist ESAT6 has potent antigenic characteristics and does not induce an allergic response. Before being docked at Toll-like receptors 2 and 4, the developed vaccine was modeled, improved, and validated. Docking studies revealed significant binding interactions, whereas molecular dynamics simulations demonstrated its stability. Furthermore, the immune system simulation indicated significant and long-lasting immunological responses. The vaccine was then reverse-transcribed into a DNA sequence and cloned into the pET28a (+) vector to validate translational activity as well as the microbial production process. The vaccine developed in this study requires further scientific consensus before it can be used against *T. pallidum* to confirm its safety and efficacy.

## 1. Introduction:

*Treponema species* are anaerobic or microaerophilic spirochetes connected to their hosts and are members of the family *Spirochaetaceae*. *Treponema subsp. Pallidum, endemicum,* and *pertenue* are the causative agents of venereal syphilis, endemic syphilis, and yaws, respectively. On the contrary, *Treponema carateum*, the causative agent of pinta, is the human pathogen most resistant to in vitro generation [1]. Until today, only three strains of *T. pallidum* causing syphilis have been entirely sequenced: Nichols [2], SS14 [3], and Chicago [4], with the ability to spread from person to person only [5]. Following acquired immunodeficiency syndrome (AIDS), syphilis is the second most fatal sexually transmitted disease. Syphilis can cause systemic diseases in adults by brutally damaging multiple organs [6]. In 1999, the World Health Organization (WHO) claimed that 12 million new cases of syphilis were detected, with more than 90% of these cases occurring in developing countries. In most of these documented cases, congenital syphilis is the leading cause of miscarriage and infant mortality [7]. Although most *T. pallidum* strains are not drug-resistant, syphilis has recently been prevalent in humans [8].

Syphilis and AIDS share the same transmission routes [9]. Syphilis disease, if left untreated, can last for many years and is divided into phases. Primary Syphilis, secondary Syphilis, and early latent Syphilis are all types of early-stage disease, while late syphilis includes late latent Syphilis and tertiary Syphilis (neurosyphilis, cardio syphilis, and gumma) [10,11]. 

Syphilis has resurrected in certain developed nations, despite the availability of advanced diagnostic tests and antibiotic therapy. In the 1990s, large-scale syphilis outbreaks in Russia and China predominantly affected heterosexuals. In contrast, fewer outbreaks occurred in homosexuals (primarily men and men) in the United States, Canada, and England [12,13]. However, recent increases in the incidence of syphilis among expected mothers in the United States suggest that heterosexually transmitted syphilis is also becoming a major concern in the United States [13]. Around 2 million new syphilis infections are recorded worldwide [8].

The fact that syphilis in an early stage (i.e., primary and secondary stages) promotes HIV transmission by 2 to 5 times, therefore, it is an active entity, facilitating the spread of HIV, and it is a crucial threat related to raising syphilis statistics [1,14]. Various reports in 1964 [15] and 1976 have reported the failure of erythromycin therapy for syphilis in pregnant women [16]. Erythromycin may not penetrate the placental barrier effectively [17].

Moreover, *T. pallidum* remains extremely susceptible to penicillin, despite the fact that it has been used to treat syphilis disease for more than seven decades. The use of azithromycin as an oral antibiotic has grown in popularity as treatment complications have increased. However, resistance to macrolides has been documented in many countries [5,18]. Various recent reviews reported that the prevalence of syphilis has increased in developed and civilized nations in the past decade, highlighting the need for an effective diagnosis of syphilis and the development of effective vaccines against syphilis.

Previous research by Zhao et al., 2011 used membrane proteins as vaccine candidates. Their findings revealed that *T. pallidum* outer membrane proteins play an essential role in *T. pallidum* virulence and are the primary target of protective immunity of the host [8,19].

Approximately 20 *T. pallidum* OMP antigens have been discovered so far. Gpd antigen [20,21], Tp92 antigen [22], and *Tpr* family antigens [23,24] have been thoroughly investigated in terms of cellular localizations, structures, functions, and gene conservation. These OMPs are believed to cause animals to synthesize opsonic antibodies, which turn these proteins into opsonin targets, followed by phagocytosis and the destruction of *T. pallidum*. Due to its high homologies between strains and relatively good immunogenicity and protective potential, antigen Tp92 [18,22,25] is the best choice for *T. pallidum* vaccination trials among the membrane proteins outlined above. The Tp92 gene of the Nichol strain is highly conserved and contains 95.5–100% sequence identity with other *Treponema* species. As a result, the Tp92 antigen may be a suitable option for vaccine research. Such vaccinations could provide a high degree of protection against the Nichols strain and other strains of *T. pallidum* [8].

Although high-throughput methods and synthetic chemistry have drastically accelerated the drug development process in recent years, it will still take almost 10–15 years to bring a new drug to market, which requires significant investment [26]. Recently, using an in silico technique to work with bacterial pathogens, many targets have been found that are drug resistant or for which no relevant vaccine is available [27]. In the post-genomic age, reverse vaccinology is a common and popular method for quickly identifying new vaccine targets [28,29]. This study aims to use reverse vaccinology and subtractive genomics, in which we are primarily interested in identifying potential vaccines and novel therapeutic targets for syphilis disease.

## 2. Materials and Methods

### 2.1. Protein Sequence Retrieval

**Figure 1:**
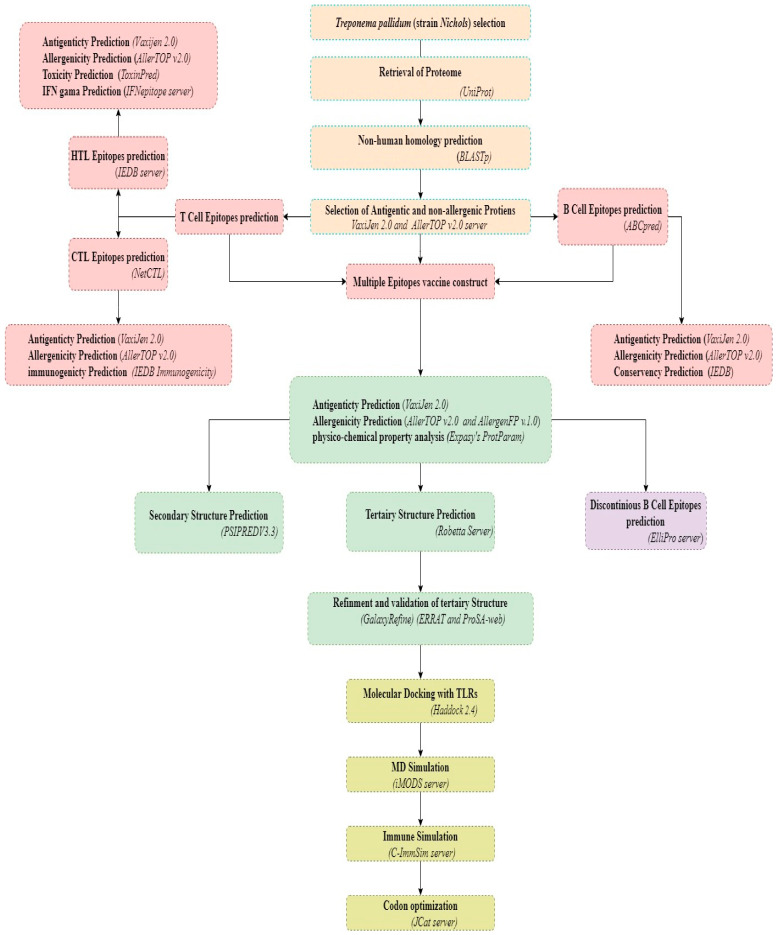
The flowchart represents the overall methodology for developing a multi-epitope subunit vaccine construct.

Figure 1 displays the sequential analysis employed in this investigation. The 1027-protein proteome of *T. pallidum* (strain Nichols) (UniProt ID: UP000000811) was obtained from UniProtKB “https://www.uniprot.org (accessed on 1 July 2022)” [30]. The new concept utilized immunoinformatics to construct a vaccine based on many epitopes. To eliminate host non-homologous proteins, pathogen proteins were aligned to the human proteome (Homo sapiens, taxonomic ID: 9606) employing the NCBI integrated BLASTp program with default parameters, except for an e-value of 10–5 “http://blast.ncbi.nlm.nih.gov/Blast/ (accessed on 1 July 2022)” [31]. Eliminating homologous host sequences is essential because these proteins generate cross-reactivity with host proteins, leading to unfavorable immunological reactions [32].

Paralogous protein with 80% sequence homology was filtered using the cluster database with high tolerance (CD-HIT) suite “http://weizhongli-lab.org/cd-hit/ (accessed on 2 July 2022)” at a sequence identity cut-off of 0.80 (http://weizhongli-lab.org/cd-hit/ (accessed on 1 July 2022)) to decrease overlap in epitope selection due to sequence similarity [33]. For additional analysis, non-paralogous and non-homologous proteins were utilized.

### 2.2. Prioritization of Essential Genes

The non-paralogous proteins were then compared to the DEG database http://tubic.tju.edu.cn/deg/ (accessed on 2 July 2022) using BLASTP at 10^−5^ to exclude non-essential genes [34]. Essential protein screening is significant because they are necessary for pathogen survival, and their deletion could inhibit cell growth, making them attractive vaccine targets [35].

### 2.3. Subcellular Localization

**Figure 2:**
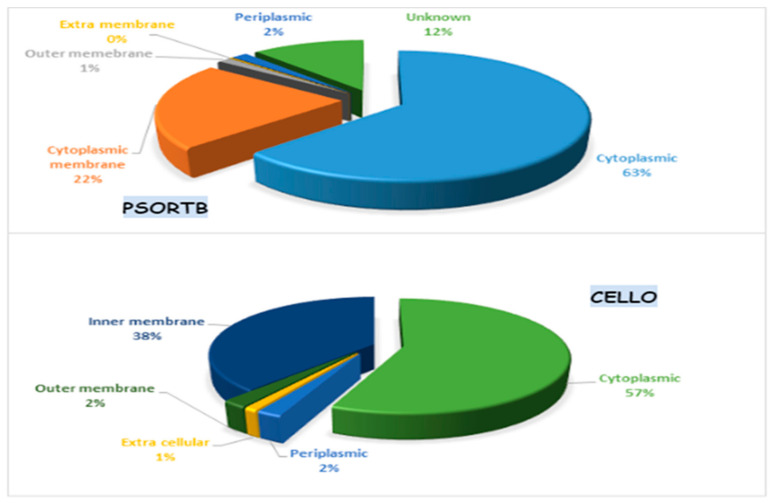
Subcellular localization of essential proteins predicted by PSORTB CELLO server. PSORTB predicted 63% cytoplasmic, 22% cytoplasmic membrane, 2% periplasmic, 1% outer membrane, 0% extra membrane and 12% unknown proteins, while Cello showed 57% cytoplasmic, 38% inner membrane, 2% periplasmic, 1% extracellular and 2% outer membrane protein.

PSORTb server “http://db.psort.org/ (accessed on 4 July 2022)” [36] and Cello server “http://cello.life.nctu.edu.tw/cello.html (accessed on 4 July 2022)” were employed to assess subcellular localization (Figure 2). The CELLO server achieved an accuracy of 88% [37] and is based on the system vector machine (SVM). The outer, extracellular, and cytoplasmic membrane proteins perform a crucial function in pathogen adherence and infection. Those categorized as extracellular and outer membranes were cross-checked utilizing CELLO2GO “http://cello.life.nctu.edu.tw/cello2go/ (accessed on 4 July 2022)” to ensure consistency in the results [38].

### 2.4. Druggability of Cytoplasmic Membrane Proteins

Likewise, the critical non-homologous cytoplasmic membrane proteins were investigated using BLASTp with an E-value of 105 against the DrugBank database “http://www.drugbank.ca/ (accessed on 5 July 2022)” to detect their drug target as well as their potential to recognize novel targets [39,40]. Comparing cytoplasmic membrane proteins to the FDA-approved customized therapeutic targets dataset. Proteins with a high similarity frequency to a DrugBank database that has been approved by the FDA were identified as therapeutic targets. New therapeutic targets that did not interact with recognized drugs or drug targets in the DrugBank were chosen for further examination.

### 2.5. Resistance Protein Analysis

The current study identified resistance proteins using the database ARG-ANNOT (Antibiotic Resistance Gene ANNOTation) database [41]. This database contains nucleotide or protein sequences resistant to multiple classes of antibiotics, including beta-lactamases, fluoroquinolones, aminoglycosides, fosfomycin, and sulfamide [42]. To identify antibiotic resistance-associated proteins, a local BLAST algorithm of cytoplasmic membrane proteins was run against antibiotic resistance sequences in ARG-ANNOT with an E-value cut-off of 10–5 in Bio-edit software.

### 2.6. Virulent Proteins Evaluation

Using BLASTp against the Virulence Factor Database (VFDB), pathophysiology and virulence variables related to disease development were preserved [43]. With an identity significantly greater than 25% and a bit score greater than 100%, these proteins were classified as pathogenic [44], making them good candidates for the development of vaccines. We chose a 25% identity cut-off since the proteins had the most significant numbers. Therefore, we needed to minimize it. This cut-off has been utilized in earlier research [45].

### 2.7. Prediction of Antigenic Proteins

Antigenicity testing was performed on the finalized virulent proteins; antigenicity was estimated utilizing VaxiJen 2.0 “http://www.ddg-pharmfac.net/vaxijen/VaxiJen/VaxiJen.html (accessed on 5 July 2022)” at a threshold of 0.4 [46]. The specified antigenic proteins were then uploaded to the online molecular weight computation tools “https://www.bioinformatics.org/sms/prot_mw.html (accessed on 5 July 2022)”. The protein with the lowest molecular weight is more appropriate for vaccine development [47].

### 2.8. Protein–Protein Interaction Network Analysis

Using the STRING protein interaction database “https://string-db.org/, (accessed on 6 July 2022)” the protein–protein interactions (PPI) database was utilized to identify potential metabolic functional links between all antigenic proteins. The STRING database aims to provide a comprehensive analysis and integration of direct (physical) and indirect (functional) protein–protein interactions [48]. Antigenic protein sequences were initially provided as input to the STRING database. Then, *Treponema pallium* subsp. *pallidum* str. The Nichols strain was then utilized as a reference strain for the PPI investigation.

### 2.9. MHC-I Binding Epitopes (CTL) Prediction Epitopes

Cytotoxic T lymphocyte epitopes (CTL) were identified with the NetCTL1.2 server “https://services.healthtech.dtu.dk/service.php?NetCTLpan-1.1 (accessed on 7 July 2022)” at 0.75 thresholds. CTL epitopes are determined on the basis of C-terminal proteasomal cleavage score, transporter associated with antigen presentation (TAP) competence, and binding of the MHC-1 complex [49]. Utilizing an artificial neural network, the MHC-I peptide binding and proteasomal C-terminal cleavage scores were determined. Furthermore, a 95-weight matrix was used to create the TAP score [49].

### 2.10. Evaluation of Predicted CTL Epitopes for Antigenicity, Allergenicity, and Immunogenicity

Using the VaxiJen v2.0 server, the antigenicity of isolated CTL epitopes was evaluated [46]. To determine the development of an appropriate immune response within the human body, the immunogenicity of these epitopes was evaluated using the IEDB Immunogenicity tool “http://tools.iedb.org/immunogenicity/ (accessed on 8 July 2022)”. Vaccine components should not induce an adverse effect. The AllerTOP v2.0 server “https://www.ddg-pharmfac.net/AllerTOP/method.html (accessed on 8 July 2022)” was employed to distinguish allergens from non-allergens in order to predict allergenicity and ensure that the vaccine construct will not produce an allergic reaction in humans [50]. The allergic and non-allergenic sequences were predicted using the k-nearest neighbor technique (kNN, k = 1). Excluded were allergenic, non-antigenic, as well as non-immunogenic epitopes.

### 2.11. MHC-II Binding Epitopes (HTL) Prediction Epitopes

For class II MHC, helper T cell lymphocyte epitopes were predicted using the Immune Epitope Database (IEDB) “http://www.iedb.org/ (accessed on 9 July 2022)” server, with a set of 7 human alleles, namely HLA-DRB1*03:01, HLA-DRB3*01:01, HLA-DRB1*15:01, HLA-DRB1*07:01, HLA-DRB4*01:01, This website predicts epitopes based on receptor affinity, computed from the IC50 value of each epitope (binding score). Typically, the IC50 value for epitopes with higher binding affinity is less than 50 nM. The IC50 number is negatively connected to the binding affinity; if the IC50 score is high, the epitope’s binding affinity to MHC-II is low, and vice versa. The percentile rank negatively correlates with epitope-MHC-II binding affinity [51]. Low percentile scored and non-overlapping epitopes were further studied for vaccine development.

### 2.12. Evaluation of Predicted HTL Epitopes for Toxicity, Antigenicity, and Allergenicity

VaxiJen 2.0 [46] was used to verify the antigenicity of the final HTL epitopes, and the AllerTOP v.2.0 server [50] was selected for allergenicity prediction. Antigenic epitopes were run through the ToxinPred server “http://crdd.osdd.net/raghava/toxinpred/ (accessed on 9 July 2022)” to determine their toxic potential. Toxic epitopes were filtered out of the analysis [52].

### 2.13. Identification of Cytokine-Inducing HTL Epitopes

HTLs release interferon-gamma (IFN-γ) cytokines that play a critical role in adaptive and innate immune responses. HTL epitopes can potentially suppress the proinflammatory response, reducing tissue damage. Therefore, the IFN-γ inducing HTL epitopes were predicted using the IFN epitope server “http://crdd.osdd.net/raghava/ifnepitope/scan.php (accessed on 9 July 2022)”. Prediction was performed using a hybrid approach of a motif and support vector machine (SVM) [53].

### 2.14. Linear B Cell Epitope Prediction and Evaluation

B cells are required to activate the humoral immune response and plasma cell production in response to a specific antigen. The ABCpred epitope server “http://ailab-projects1.ist.psu.edu:8080/bcpred (accessed on 10 July 2022)” was used to predict linear B cells (each of 20 lengths) with an accuracy of 75% (0.49 sensitivity and 0.75 specificities). This server investigated two machine learning algorithms to predict flexible-length linear B-cell epitopes. The first method uses sequence kernels to calculate a similarity score between two variable-length sequences. The second technique employs a variety of mapping algorithms to convert a variable-length sequence into a fixed-length feature vector [49]. We also used VaxiJen 2.0, AllerTOP v2.0 servers, and the IEDB server conservancy “http://tools.iedb.org/conservancy/ (accessed on 10 July 2022)” to analyze antigenicity allergenicity and conservancy to rank and shortlist epitopes [54].

### 2.15. Discontinues the Prediction of the B Cell Epitope

The multi-epitope 3D structure was refined and validated and then uploaded to the ElliPro server “http://tools.iedb.org/ellipro/ (accessed on 11 July 2022)” [55]. This server is based on the theory that antibodies are better able to bind to exposed regions of a protein. Treating the protein as an ellipsoid allows us to locate these protruding residues. Researchers have discovered that 90% of B-cell epitopes are discontinuous [54] and that these epitopes are made up of residues that are physically far apart in the basic structure but are brought close together by protein folding. The PI (Protrusion index) value and clustering approaches, i.e., the distance R between the center of mass of the residues, are used to determine discontinuous epitopes. Larger predicted discontinuous epitopes correlate with longer residues [54,56].

### 2.16. Assembling of Vaccine Construction Final Multi-Epitope

The final vaccine construct contains 4 CTL, 4 HTL, 11 LBL epitopes, and the adjuvant. Toll-like receptor 2 (TLR2) agonist ESAT6 (Accession: AEP68523.1) was taken as an adjuvant [57]. ESAT6 stimulates the secretion of IL-6 and TGF-β by dendritic cells in a TLR2-dependent way; it also induces Th17 immune responses, which are essential for optimal vaccine efficacy [58]. ESAT6 generated by an *E. coli* expression system increased IFN- gene expression [59]. The selected epitopes were fused with the help of specific peptide linkers. Each CTL epitope was a linker through the AAY linker [60], HTL epitopes fused with the GPGPG linker [57], and each LBL epitope was joined using KK linkers [61]. The EAAK linker was used to attach the adjuvant with CTL epitopes to the N-terminal of the vaccine construct [62].

### 2.17. Evaluation of the Physicochemical Properties, Antigenicity, and Allergenicity of the Vaccine Construct

After the vaccine construct was designed, the number of physicochemical parameters was determined using Expasy’s ProtParam “https://www.expasy.org/ (accessed on 12 July 2022)” [63]. These parameters included the vaccine’s molecular weight, theoretical isoelectric point (pI), total number of positive and negative residues, extinction coefficient, instability index, half-life, aliphatic index, and grand average hydropathy (GRAVY). The antigenicity of the finalized vaccine design was analyzed with Vaxijen v2.0 [46]; the allergenicity was computed with AllergenFP v.1.0 “https://ddg-pharmfac.net/AllergenFP (accessed on 12 July 2022)”, and both programs were updated to their most recent versions [64].

### 2.18. Prediction of the Secondary and Tertiary Structure of the Vaccine Design

Using the PSIPREDV3.3 web server “http://bioinf.cs.ucl.ac.uk/psipred/ (accessed on 13 July 2022)”, the two-dimensional (2D) structural modeling of the intended construction was anticipated. The PSIPRED server uses the query amino acid residues in conjunction with two-feed-forward neural networking as well as PSI-BLAST to generate a 2D model. The three-state accuracy (Q3) of the server was 81.6% [65].

The three-dimensional (3D) model of the designed vaccine construct was obtained through Robetta Server “http://robetta.bakerlab.org (accessed on 13 July 2022)”. The vaccine sequence was parsed into putative domains, and the server used either a de novo strategy for structure prediction or a comparative modeling technique to build a 3D structure. The server looks for suitable templates for comparison modeling and utilizes them. If the desired amino acid sequence template is missing, the server uses the de novo Rosetta fragment insertion method [66].

### 2.19. Refinement and Validation of 3D Structure

Unfortunately, it is possible that the predicted 3D structures of proteins using computational methods do not precisely match their natural structures. The 3D structure was refined to improve its resolution from the first low-resolution prediction. The predicted model was refined utilizing GalaxyRe-fine “http://galaxy.seoklab.org/cgi-bin/submit.cgi?type=REFINE (accessed on 15 July 2022)”. To relax the overall structure, this server uses MD simulation after first reconstructing the side chains and repacking them [67].

Finally, the ProSA-web server “https://prosa.services.came.sbg.ac.at/prosa.php (accessed on 16 July 2022)” was used to verify the accuracy of the 3D models. Analyzing the model’s atomic coordinates, ProSA-web calculates an overall quality score. The ProSA-web z-score is a graphical representation of the z-score of structures obtained experimentally and submitted to PDB [68]. Nonbonded atom-atom interactions in the vaccine 3D structure were analyzed using the ERRAT server “https://saves.mbi.ucla.edu/ (accessed on 16 July 2022)” [69]. The Ramachandran plot was obtained using the PROCHECK service “https://saves.mbi.ucla.edu/PROCHECK/ (accessed on 16 July 2022)”. The dihedral angles psi (ψ) and phi (Φ) of the amino acids are used to illustrate their energy acceptability and prohibition, respectively, in the Ramachandran plot, which uses the van der Waals radius of the side chain. The PROCHECK results have included the fraction and the total number of residues in the most preferred, generously allowed, extra allowed, and forbidden areas, which characterize the quality of the modeled construction [70].

### 2.20. Molecular Docking of Constructed Vaccine with TLR2 and TLR-4

To evaluate the interaction between a protein and its receptor, scientists have developed a computational method called molecular docking [71]. The structures of the TLR 2 and TLR 4 receptors (PDB ID: 6ING and PDB ID: 2Z63, respectively) were downloaded from the Protein Data Bank (http://www.rcsb.org/, accessed on 18 July 2020) to use as a docking partner for the improved vaccination constructions. The HADDOCK server “https://wenmr.science.uu.nl/haddock2.4/ (accessed on 18 July 2020)” conducted the docking. HAD-DOCK uses ambiguous interaction restraints (AIRs) to encode data from known or predicted protein interactions and drive the docking procedure. Furthermore, it enables the definition of clear, granular distance constraints (e.g., from MS cross-links). It reinforces several other types of experimental information, such as residual dipolar couplings from NMR, pseudo-contact shifts, and cryo-EM maps. Accordingly, the HADDOCK refinements server was applied to a typical docked structure with a lower score [71]. The refined TLR-vaccine structures were visualized in PyMol. PDBsum was also utilized to generate a visual representation of vaccine–TLR-4 interaction residues [72].

### 2.21. Molecular Dynamics Simulation

We performed a molecular dynamics simulation to analyze the stability of the vaccine–receptor complex [73,74]. To study the molecular behavior and assess the stability of the protein–ligand complex, a molecular dynamics simulation was applied, as it provides an overview of the physical basis of the complex analyzed [75]. The iMODS server “https://imods.iqfr.csic.es/ (accessed on 21 July 2022)”, which explores the collective motions of proteins using normal mode analysis in internal coordinates, was employed for this purpose, and the parameters were kept as default in the server. The server evaluated the stiffness, deformability, B-factors, and covariance of each complex residue. The eigenvalue shows the motion rigidity of the structure, while the deformability plots reveal the non-rigid sections. The lower the eigenvalue, the more easily the complex may be deformed since the energy required to distort it is proportional to the eigenvalue.

### 2.22. Immune Simulation

Using C-IMMSIM v10.1, the immunological responsiveness to the constructed vaccine was simulated. Using the C-ImmSim server accessible at “https://kraken.iac.rm.cnr.it/C-IMMSIM/ (accessed on 23 July 2022)”, immunological simulations were performed in silico to assess the immunogenic significance of the final vaccine. This immunity simulator uses machine learning techniques and a particular scoring matrix (PSSM) [76]. As previously described by Castiglione et al. [77], we used a minimum delay of 28 days between two doses. A total of three injections were provided in silico with time steps of 1, 84, as well as 170, where the one-time step corresponds to 8 h in reality. The maximum value for the simulation steps was adjusted to 1050.

### 2.23. Codon Optimization of Vax Sequence and In Situ Cloning

When attempting to express a foreign gene in a host organism, it is often necessary to optimize the codons used. The Java Codon Adaptation Tool “https://www.prodoric.de/JCat/ (accessed on 25 July 2022)” was employed for reverse translation as well as codon optimization of the specified vaccine constructions to optimize the vaccine construct in the *E. coli* K12 strain for maximal protein expression [78]. Three excess choices were chosen to achieve the intended result: a prokaryote ribosome binding site, restriction enzyme cleavage, and rho-independent transcription termination. The server delivers the codon adaptation index (CAI) (>0.8–1.0) and the GC content (30–70% in the output files to verify the transcription and translation efficiency of the planned vaccine construct [79]. Two restriction sites, EcoRI and BamHI, were inserted into the C and N terminals of the sequence, and SnapGene v5.0.8 software was employed to clone the modified sequence with the restriction site into pET-28a (+) plasmid obtained from SnapGene “https://www.snapgene.com/ (accessed on 21 July 2022)” [80].

## 3. Results

### 3.1. Proteome Collection

Various immunoinformatic and subtractive proteomic approaches were used to design multiple epitope vaccines to protect against infection caused by *T. pallidum* [31]. UniProtKB was used to extract the whole reference proteome of the *T. pallidum* strain Nichols with 1027 proteins in FASTA format (UniProt ID: UP000000811).

### 3.2. Removal of Homologous Proteins

Specificity filter against the human proteome (taxonomic ID: 9606) using NCBI BLASTP “https://blast.ncbi.nlm.nih.gov/Blast.cgi (accessed on 1 July 2022)” with a cut-off value of 10–5 against the proteome of the hole of *T. pallidum*. Because the target and host proteins are cross-reactive, homologous proteins to the host produce an autoimmune reaction [33]. The remaining 983 proteins were recognized as non-homologous and hired for further investigation.

### 3.3. Prediction of Paralogous Proteins

The CD-Hit server “http://weizhongli-lab.org/cdhit/ (accessed on 2 July 2022)” was utilized to exclude paralogous sequences with an identity of at least 80% from non-human proteins. These sequences do not make a significant contribution to the survivability of organisms. Therefore, excluding them from the core proteome is prudent. The CD-Hit server identified 979 non-paralogous proteins (99.59%) and only four paralogous proteins (Table 1). The non-paralogous proteins were transferred to the subsequent production phase.

### 3.4. Essential Proteins Prediction

The non-paralogous proteins were run against DEG to determine essential proteins for *T. pallidum* survival using BLASTp at the 10^−5^ cut-off value. The results of BLASTp characterized 476 proteins as essential for *T. pallidum* survival. Non-essential proteins were excluded.

### 3.5. Subcellular Localization of the Essential Proteins

Using the PSORTb server for the localization of remaining essential proteins in the cell, the server localized the proteins based on their location as; 300 Cytoplasmic, 105 cytoplasmic membranes, six outer membranes, one extracellular, eight periplasmic, and 56 unknown proteins and another software “Cello” server localized the protein as; 321 cytoplasmic, 92 inner membranes, 30 outer membranes, 26 periplasmic, seven extracellular [38]. Figure 2 shows that surface proteins such as outer, extracellular, and cytoplasmic membranes are associated with pathogenicity, helping adhere to pathogens, invasion, proliferation of host tissue, and ultimately successful survival. Targeting these proteins is more suitable for vaccine design [44].

### 3.6. Druggability of Cytoplasmic Membrane Proteins

Only 15 of 95 cytoplasmic membrane proteins demonstrated interaction druggability potential with FDA-approved medications, according to the DrugBank database. All fifteen of these proteins potentially act as therapeutic targets in developing antibiotics against this infection. In addition, the remaining 80 that had no similarity to any recognized therapeutic targets in the DrugBank database were declared novel therapeutic potential targets. Consequently, only these proteins underwent additional investigation.

### 3.7. Resistance Protein Analysis

The resistance protein involved in the resistance process might be used as a therapeutic target. Using Bioeditor software, the list of cytoplasmic membrane proteins was then BLAST against the ARG-ANNOT database. The ARG-ANNOT database identified approximately 12 cytoplasmic membrane proteins were identified by the ARG-ANNOT database; these proteins are responsible for inducing antibiotic resistance in *T. palladium.*

### 3.8. Virulent Protein Analysis

Virulent protein prediction is crucial, and these proteins allow bacterial pathogens to bypass host immune responses. BLASTp screening of cytoplasmic membrane proteins against VFDB (Virulence Factor Database) identified nine proteins; Membrane lipoprotein TpN32 (UniProt ID: O07950), Uncharacterized periplasmic metal-binding protein (UniProt ID: O83077), DNA translocase FtsK (UniProt ID: O83964), Lipoprotein-releasing system ATP-binding protein (UniProt ID: O83590), Protein Soj homolog (UniProt ID: O83296), Site-determining protein (UniProt ID: F7IVD2), amino acid ABC transporter, ATP-binding protein (UniProt ID: F7IVD2), ABC transporter, ATP-binding protein (UniProt ID: O83930) and Sugar ABC superfamily ATP-binding cassette transporter, ABC protein (UniProt ID: O83782) proteins as virulent with >25% identity and 100% bit-score, and selected for future investigation [42].

### 3.9. Vaccine Protein Prioritization

The virulent proteins were subjected to the Vaxijen server to predict antigenic potential. Out of nine virulent proteins, six proteins were shortlisted; Membrane lipoprotein TpN32 (UniProt ID: O07950), DNA translocase FtsK (UniProt ID: O83964), Protein Soj homolog (UniProt ID: O83296), F7IVD2 site-determining protein (UniProt ID: F7IVD2), ABC transporter, ATP-binding protein (UniProt ID: O83930) and the sugar ABC superfamily ATP-binding cassette transporter, ABC protein (UniProt ID: O83782) having high antigenic score as; 0.5303, 0.4699, 0.4275, 0.4611, 0.5249 and 0.5621 were predicted by Vaxijen server at threshold 0.4. The molecular weight of the protein is one of the most important parameters in vaccine design. A protein with the least molecular weight can be efficiently purified during the subsequent validation process. An online protein molecular weight server was used to predict the molecular weight of proteins. The molecular weight of the membrane lipoprotein TpN32 protein was 29.09 kDa, the DNA translocase FtsK protein was 86.62 KDa, the Soj homolog protein was 27.35 KDa, the site-determining protein was 33.72 ABC transporter, the ATP-binding protein was 25.18 KDa and the Sugar ABC superfamily ATP-binding cassette transporter, the ABC protein was 42.65 KDa, respectively, and thus strongly consider future vaccine development.

### 3.10. Protein–Protein Interaction Network Analysis

**Figure 3:**
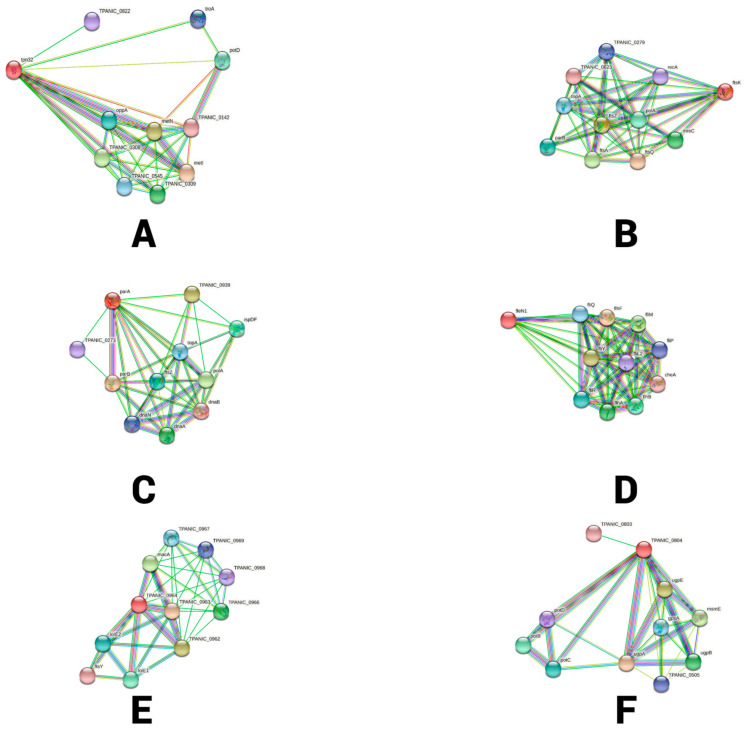
The antigenic proteins and protein interaction (PPI) through the STRING database. (**A**) Protein 1: 007950|TPN32_TREPA Membrane lipoprotein TpN32; (**B**) Protein 2: O83964|FTSK_TREPA DNA translocase FtsK; (**C**); Protein 3: O83296|SOJ_TREPA Protein Soj homolog (**D**); Protein 4: F7IVD2_TREPA site-determining protein; (**E**) Protein 5: O83930_TREPA ABC transporter, ATP-binding protein; (**F**) Protein 6: O83782_TREPA Sugar ABC superfamily ATP-binding cassette transporter, ABC protein.

The antigenic proteins were subjected to STRING database for a protein–protein interaction study. STRING database revealed that the membrane lipoprotein TpN32 shows interaction with ten proteins, such proteins are metN (Methionine abc superfamily), potD (Spermidine/putrescine abc superfamily), troA (Periplasmic zinc-binding protein), metI, oppA, TPANIC_0545, TPANIC_0308, TPANIC_0309, TPANIC_0822 and TPANIC_0142 (uncharacterized) (Figure 3A). Figure 3B reveals that the FtsK DNA translocase interacts with ftsQ, ftsZ, ftsA (cell division protein), mreC (Cell shape determining protein), polA (DNA-directed DNA polymerase), parB (chromosome partitioning protein), topA (DNA topoisomerase), recA (recombination protein 7), TPANIC_0623 and TPANIC_0279 (uncharacterized). Protein Soj homolog (SOJ_TREPA) shows a connection with parB (chromosome partitioning protein), polA (DNA-directed DNA polymerase I), dnaA (DNA-directed DNA replication initiator protein), ispDF (2-C-methyl-D-erythritol 4-phosphate cytidylyltransferase), ftsZ (essential cell division protein), topA (DNA topoisomerase topa), dnaN (confers DNA tethering and processivity to DNA polymerases and other proteins), dnaB (replicative DNA helicase) and uncharacterized TPANIC_0273 and TPANIC_0939 proteins (Figure 3C). Figure 3D shows that the site-determining protein F7IVD2_TREPA interacts with flhF, fliY, fliM flhA, flhB, fliR, fliQ, flip, fliL2, and cheA. Of these, flhA, flhB, fliR, fliQ and flip are (virulence-related) secretory pathway proteins that belong to the type iiisp family iii; similarly, fliY and fliM are flagellar motor switch proteins, fliL2 is a flagellar basal body-associated protein that controls the rotational direction of flagella during chemotaxis. flhF is a flagellar-associated gtp-binding protein, and cheA (Sensor histidine kinase) is involved in transmitting sensory signals. Figure 3E exhibits that ABC transporter, ATP-binding protein interacted with TPANIC_0966, TPANIC_0967, TPANIC_0968 (Hypothetical protein), TPANIC_0969 (Putative outer membrane protein), ftsY (Sec family Type I general secretory pathway protein), TPANIC_0963, TPANIC_0962, macA, lolE1, and lolE2 (Uncharacterized proteins). Figure 3F shows that the Sugar ABC superfamily ATP-binding cassette transporter, ABC protein was found with interaction with potB potC potD (Spermidine/putrescine abc superfamily ATP-binding), gpsA (glycerol-3-phosphate dehydrogenase), ugpA, ugpE, msmE, ugpB, TPANIC_0505 and TPANIC_0803 (uncharacterized proteins).

### 3.11. Selection and Evaluation of T-Cell Epitopes

For each virulent protein, the NetCTL 1.2 predicted 40 CTL epitopes (9-mer). The antigenicity, allergenicity, as well as immunogenicity scores of the epitopes were also calculated. Only four of the 40 epitopes were chosen for vaccine development because they met the criteria of being non-allergenic, highly antigenic, and immunogenic (Table 2). According to a reference set of seven human HLAs, the IEDB server also predicted HTL epitopes (15-mer). Only four epitopes were predicted to have the ability to act as antigenic and nonallergenic. They were also nontoxic and could induce IFN-γ were selected for additional investigations (Table 3).

### 3.12. Selection and Evaluation of B-Cell Epitopes

The ABCpred epitope server was used to predict linear B cells (each of 20 lengths) with a precision of 75%. The ABCpred server identified a total of 22 linear B-cell epitopes; Only 11 B-cell epitopes were chosen for vaccine constructs based on their evaluated properties as non-allergenic, antigenic, and high conservancy properties (Table 4).

### 3.13. Epitope-Based Subunit Vaccine Construct

**Figure 4:**
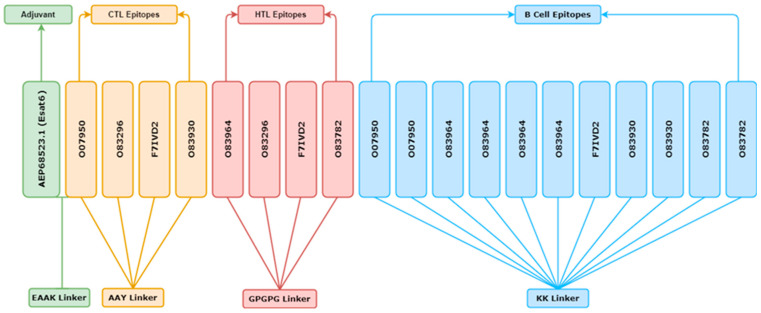
EAAAK, AAY, GPGPGP and KK linkers were used to create the final vaccine candidate configuration, consisting of an adjuvant accompanied by cytotoxic T lymphocyte (CTL), Helper T lymphocyte (HTL), and B cell epitopes.

The selected epitopes were fused with a specific linker to design a vaccine construct of multiple epitopes. It was joined by 4 CTL epitopes, while GPGPG joined 4 IFN inducer HTL epitopes, and a KK linker was used to fuse 11 LBL epitopes, respectively. To improve immunization and epitope effectiveness, the N-terminal of the vaccine construct was linked to the TLR-2 agonist ESAT6 using an EAAAK linker. The final constructed vaccine is 460 amino acid residues long (Figure 4).

### 3.14. Antigenicity and Allergenicity Physicochemical Properties of the Vaccine Construct

The model was constructed, and then its allergenicity and antigenicity were determined. According to our findings, the created model is highly antigenic (scoring 0.7878 at a 0.0.4 threshold on the Vaxijen server) and non-allergenic (as predicted by AllerTOP v2.0 [52] and AllergenFP v.1.0 [62]). The ProtParam software was then used to analyze the physiochemical properties of the constructed vaccine. The theoretical pI and GRAVY (Grand average of hydropathicity) of the generated vaccine were found to be 9.50 and −0.420 (negative sign indicates hydrophilic nature), respectively, and its molecular weight was determined to be 48,782.81 kD. With an instability index of 34.31, the constructed system is stable within the host environment. The vaccine construct’s aliphatic index was 77.09, which guarantees its thermostability. The half-life of the vaccine is approximately 10 min in yeast cells and greater than 10 h in *E. coli* (in vivo).

### 3.15. Analysis of Secondary Structure

**Figure 5:**
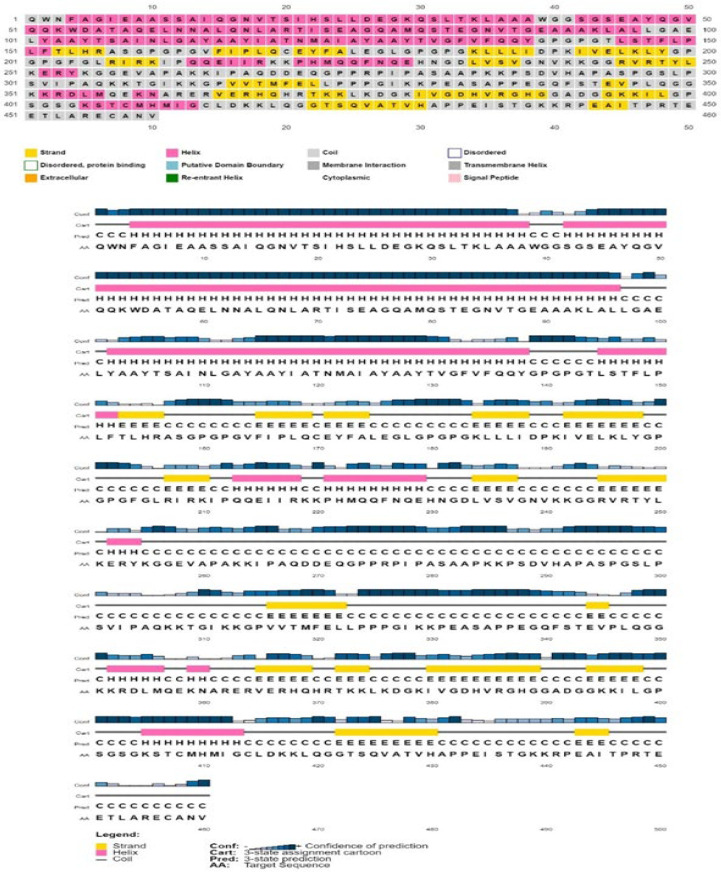
Secondary structural features of the constructed vaccine. Herein, α-helix (36.74%), β-strands (45.22%), and random coils (18.04%) are represented with pink, yellow, and blue colors, respectively.

The PSIPRED server was utilized to investigate the secondary vaccine structure. According to this server, 208 (45.22%) amino acids in the entire vaccine formed extended Beta strands,169 (36.74%) amino acids were found in alpha helixes, and 83 (18.04%) amino acids formed coils, as shown in Figure 5 [65].

### 3.16. Tertiary Structure Prediction, Refinement, and Validation of Design Vaccine

**Figure 6:**
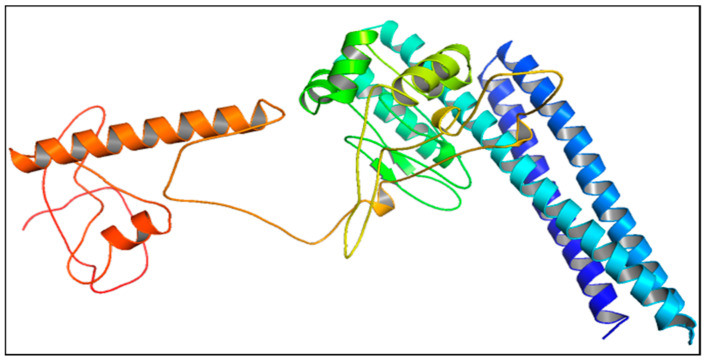
Predicted 3D structure of *T. pallidum* vaccine constructs, visualized by Pymol software.

**Figure 7:**
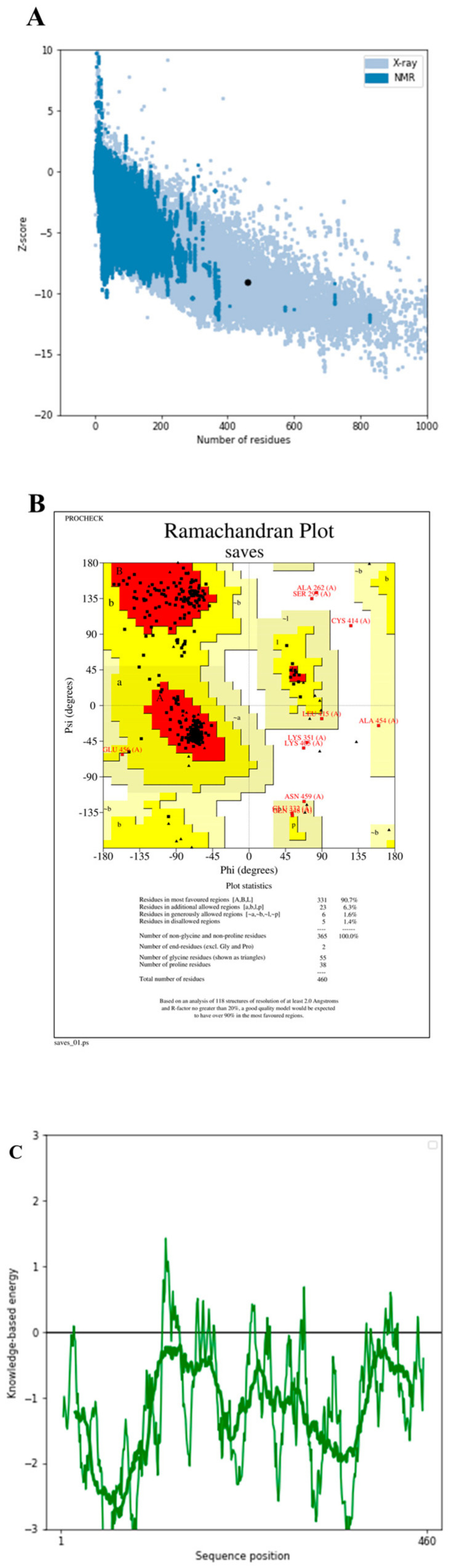

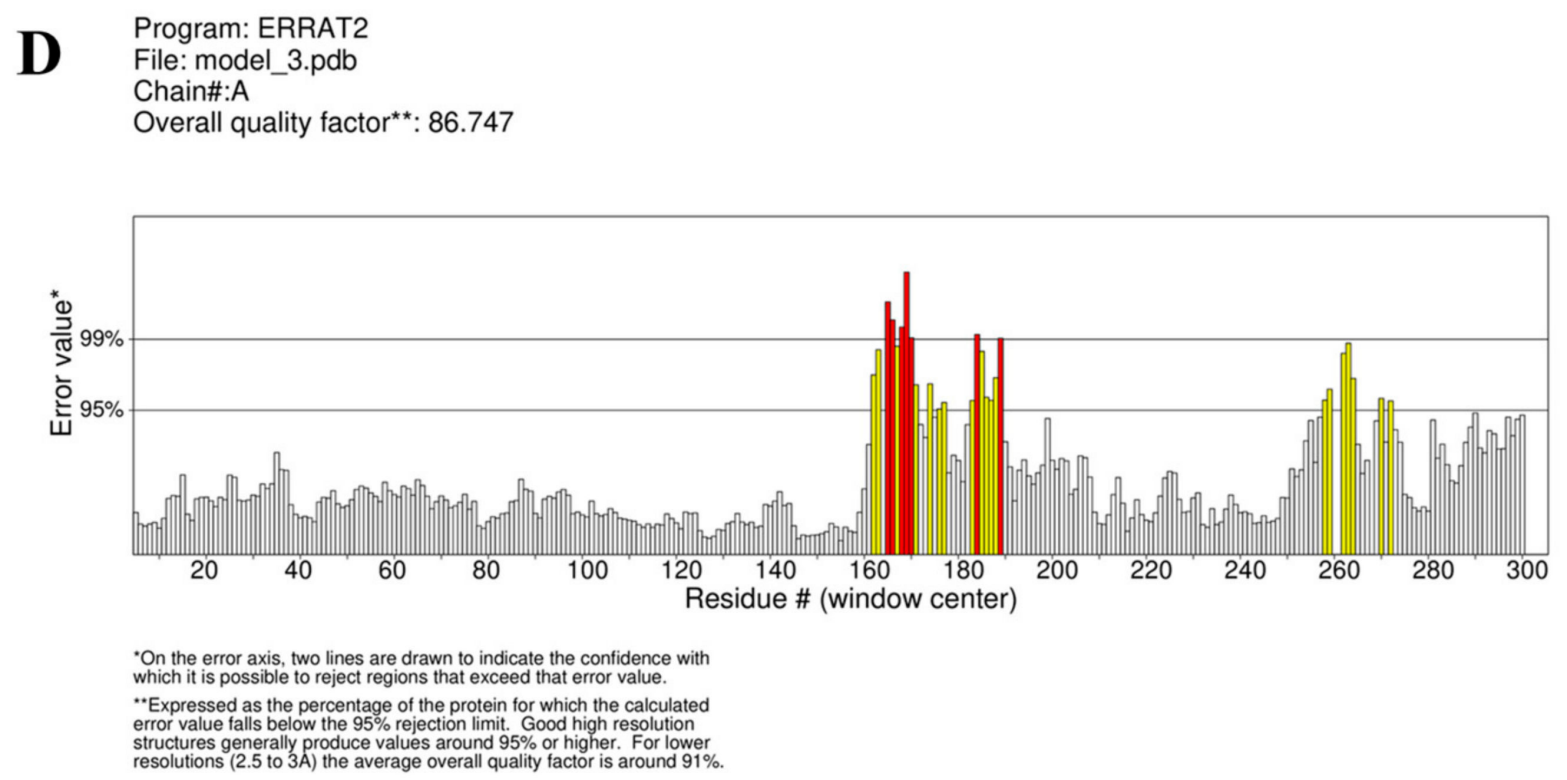
Validation of the three-dimensional structure of the vaccine constructed. (**A**) ProSA web evaluation of the vaccine structure (Z-scores −9.1); (**B**) investigation of the protein structure using the Ramachandran plot following molecular optimization; (**C**) ProSA graphical plot (local model quality); (**D**) ERRAT server predicted the overall quality of the 3D structure.

The 3D structures of the vaccine constructions were managed by the Robetta server. For any query peptide provided, the system generates five predicted configurations. A thorough evaluation led to the selection of model 2 for further study (Figure 6). The GalaxyWEB server’s GalaxyRefine module was then used to further improve the 3D structure. It was assumed that the ER-RAT, ProSA-web, and PROCHECK servers would be used to investigate and correct any structural flaws. The improved model received a Z score of −9.1 from ProSA-web, which is above the mean Z score for similar natural proteins (Figure 7A). Energy as a function of amino acids in the protein structure was another way in which Prosa-web proved the correctness of the local model (Figure 7C). Ramachandran analysis was performed using the PROCHECK service, which verified 90.7% of residues in the red region (most favorable), 6.3% of residues in the yellow region (additional allowances), and 1.6% of residues in the pale yellow area (generous allowances). A 1.4% of its residues are located in forbidden locations (highlighted in white) Figure 7B. According to the ERRAT server, the 3D structure of the vaccine has an overall quality of 86.7% (Figure 7D).

### 3.17. Molecular Docking of the Constructed Vaccine with Human TLR-2 and TLR-4

**Figure 8:**
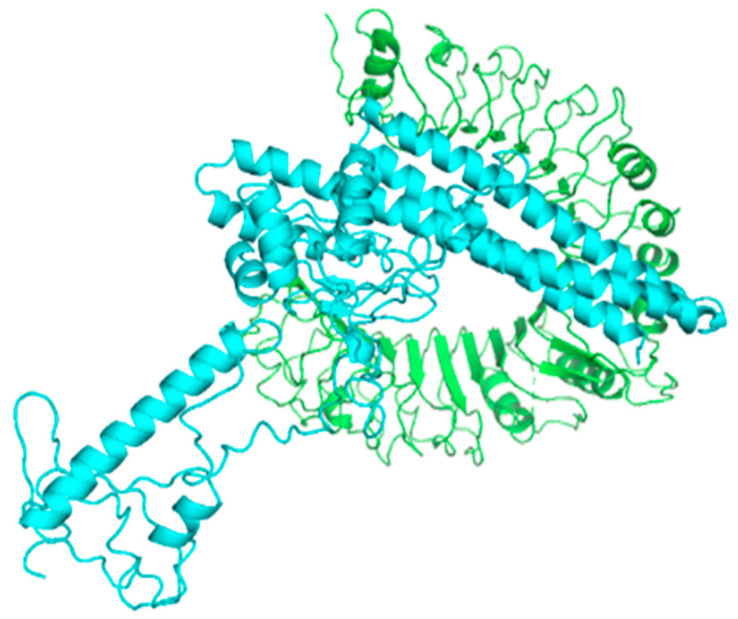
The docked complex between the designed vaccine constructs and the TLR2 receptor. Vaccine, the surface is represented in cyan, while the TLR-2 receptor is represented in green.

Using the HADDOCK server, the molecular docking of vaccine constructs was performed with human TLR-2 and TLR-4. In the case of TLR-2(6ING) docking, HADDOCK clustered 130 structures in 18 clusters, representing 65% of the water-refined models generated by HADDOCK as shown in Figure 8. The top-ranked cluster with the lowest Z-score is the most significant for docking analysis. The lowest HADDOCK score is −52.2 +/−6.4, and Z-score −2.1 is the most reliable among all clusters, and it suggests that the vaccine structure and TLR-2 interact appropriately. The creation of an excellent quality docked complex is indicated by a lower RMSD value of the docked complex. Table 5 illustrates the electrostatic, solvation, restraints violation, and van der Waals energies, in addition to Z-Score values computed by the HADDOCK. Figure 8 reveals the Pi and hydrogen interaction between vaccination and TLR-2. Analysis of the vaccine–TLR complex revealed that LYS422 binds to the benzene ring of HIS22 by Pi bond with a 5.0 Å distance. The hydrogen bonds were found between SER27 to GLU194 at 3.27 Å, SER39 to ARG156 at 2.99 Å, SER42 to GLU194 at 2.63 Å, ASN61 to LYS196 at 3.11 Å/2.78 Å, ARG321 to GLY6/GLN1 at 2.96 Å/3.26 Å, TYR323 to ASN3 at 3.26 Å, LYS347 to SER11/GLN15 at 3.03 Å/2.64 Å, LEU399 to ARG70 at 3.04 Å/2.87 Å, LYS422 to GLN66 at 2.85 Å, SER424 to ASN63 at 2.72 Å, ARG447 to GLN59 at 2.84 Å, LYS488 to GLN52 at 2.67 Å and LYS561 to ALA37 at 2.69 Å, respectively. The interaction was revealed through the PDBsum online server and PyMol software.

**Figure 9:**
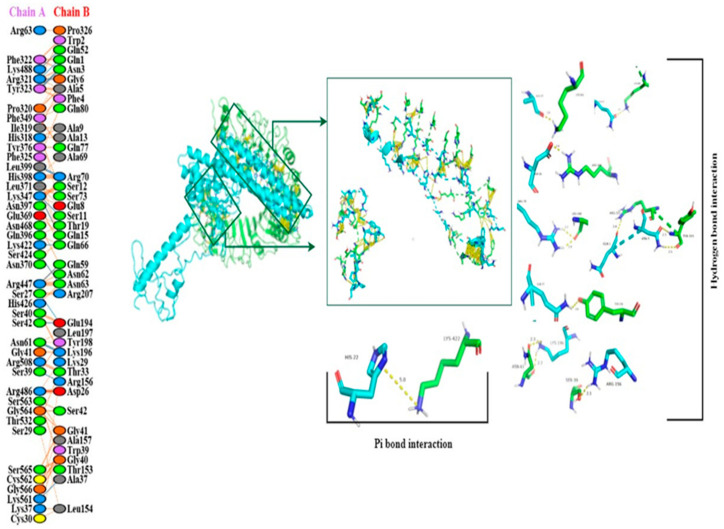
The figure shows the binding interaction between active residues of a docked complex of human TLR-2 and vaccine construct. Chain A represents active residues of TLR-2, while chain B represents active residues of the vaccine construct.

As required, a representative model from this top cluster was refined. The HADDOCK refinement server grouped the 20 generated structures into a single cluster, indicating 100% of the HADDOCK created by the water-refined model. The HADDOCK score is −250.0 +/− 4.6 with a 0.0 Z-score; however, the buried surface area (BSA) score for this refined cluster is 4493.9 +/− 92.1 (Figure 9).

**Figure 10:**
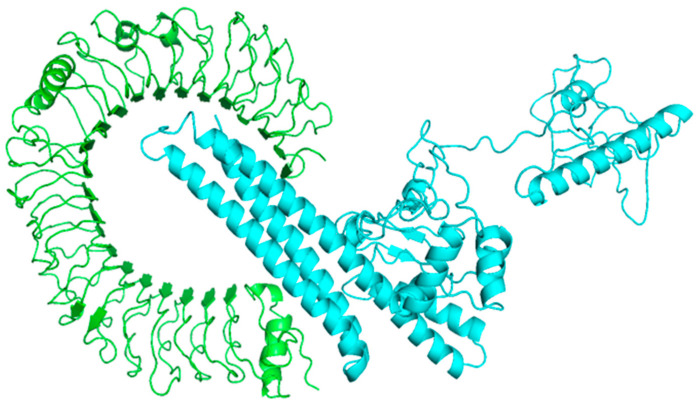
Toll-like receptor 4 (TLR4) complex docked with vaccine constructs. The Cyan color represents the vaccination surface, and the green color represents the TLR-4 receptors.

HADDOCK grouped 135 structures into 14 clusters for TLR4 (2z63) docking, representing 67% of the water-refined models developed by the HADDOCK cluster Figure 10. The cluster with the lowest HADDOCK score, 17.7 +/− 17.5, is the most reliable. The BSA score and Z-score for this docking are 2678.3 +/− 408.6 and −1.0, respectively. A decrease in the RMSD value of the docked complex indicates the development of a high-quality complex. Table 6 displays the electrostatic, solvation, restraints violation, van der Waals energies, and Z score values calculated by the HAD-DOCK software.

**Figure 11:**
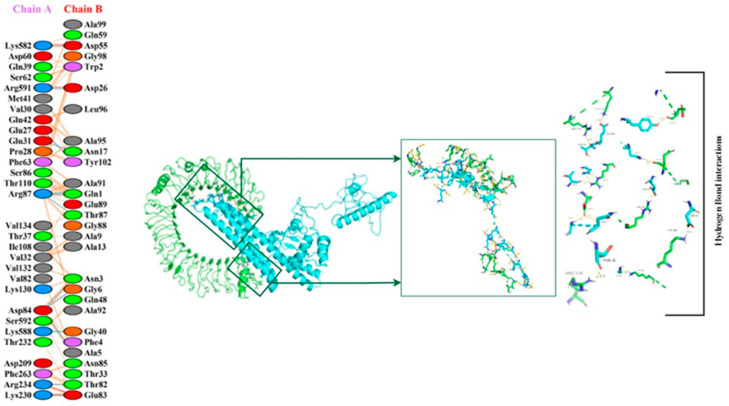
The figure shows the binding interaction between the active residues of the docked complex of human TLR-4 and the vaccine construct. Chain A represents active residues of TLR-4, while chain B represents active residues of the vaccine construct.

Figure 11 displays the molecular interaction (hydrogen bonds) between vaccination and TLR-4; GLU27 bind to TYR102 at 2.83 Å distance, GLU31 to ASN17 at 3.19 Å, ASP84 to ASN3/ALA5 and GLY6 at 3.02 Å/3.14 Å and 2.73 Å, ARG87 to GLN1 at 2.60 Å/2.91 Å, ARG87 to THR87 at 3.03 Å, LYS230 to GLU83 at 2.69 Å, ARG234 to THR82 at 2.66 Å, LYS582 to ASP55 at 2.60 Å, LYS588 to GLY40 at 3.11 Å, ARG591 to ASP26 at 2.70 Å, respectively.

The refined representative model of this top cluster was implemented as required. All of the HADDOCK models that were refined in water were included in a single cluster of 20 structures created by the server. This refined cluster has a HADDOCK score of −135.8 +/− 3.5, a buried surface area score of 2543.7 +/− 92.8, and a Z-score of 0.0.

### 3.18. MD Simulation

**Figure 12:**
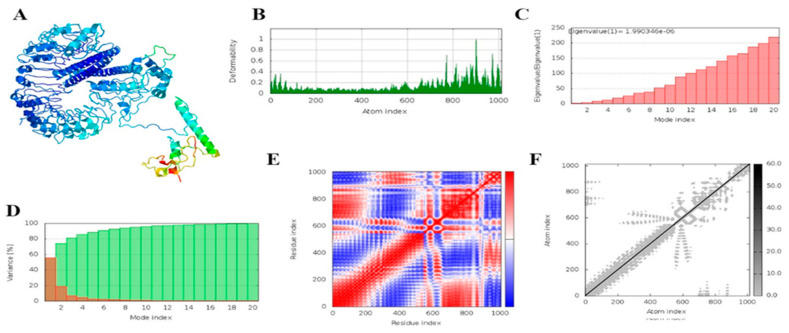
Outcomes of MD simulations of TLR-2 and the designed vaccine construct. (**A**) NMA mobility; (**B**) deformability plot; (**C**) eigenvalue plot; (**D**) variance plot (individual variances are red, while cumulative variances are green); (**E**) covariance map [correlated (red), uncorrelated (white), or anti-correlated (blue) motions]; (**F**) elastic network (darker grey regions indicate stiffer regions) of the complex.

**Figure 13:**
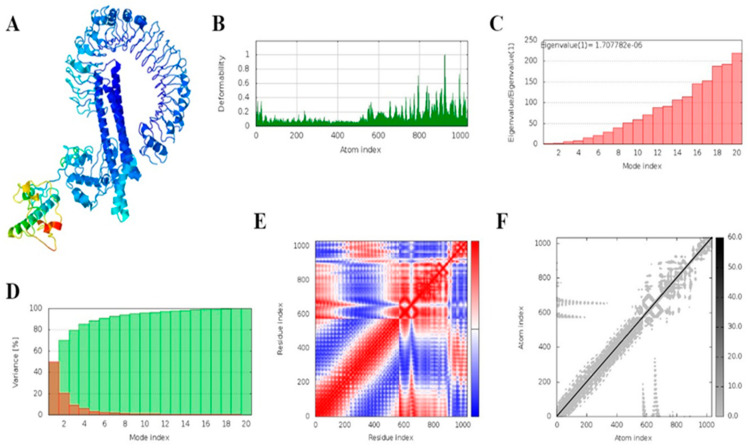
Outcomes of MD simulations of TLR-4 and the designed Vaccine construct. (**A**) NMA mobility, (**B**) deformability plot, (**C**) eigenvalue plot, (**D**) variability plot (individual variances are brown, while cumulative variances are green), (**E**) covariance map [correlated (red), uncorrelated (white), or anti-correlated (blue) motions], (**F**) elastic network (darker grey regions indicate stiffer regions) of the complex.

The iMODS server performed a molecular dynamics simulation of the docking complexes. This server uses normal mode analysis. Figure 11 and Figure 12 illustrate the simulation results for both vaccine–TLR2 and vaccine–TLR4. Figure 12 and Figure 13B demonstrate the deformability plot of both complexes, respectively, where the peaks indicated the non-rigid regions of the complexes. Eigenvalues values of vaccine–TLR2 and TLR4 docking complexes were 1.990346e^−6^ and 1.707782e^−6,^ respectively, shown in Figure 12C and Figure 13C. (Figure 12 and Figure 13D) show the variance matrix graph of residues, which are inversely related to eigenvalue, where red indicates individual variance and green is a cumulative variance. (Figure 12E and Figure 13E) reveal that the covariance matrix signifies coupling between pairs of residues, red represents experience correlated, white represents uncorrelated, and blue color shows anti-correlated motions. (Figure 12F and Figure 13F) show an elastic network of the complexes, where dots indicate one spring and a gray area indicates stiffer springs. The overall analysis of iMODS suggests that vaccine constructs with TLR2 and TLR4 complexes are stable.

### 3.19. Discontinuous B-Cell Epitope Prediction

**Figure 14:**
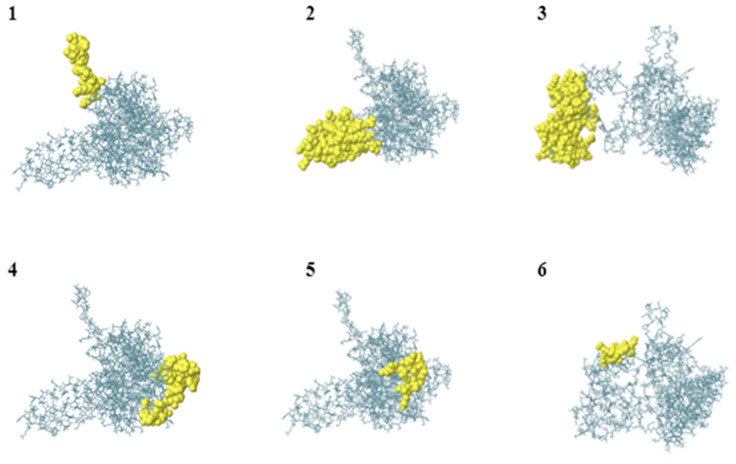
ElliPro predicted discontinuous B-cell epitopes. (**1**–**6**): 3D visualization of conformational or discontinuous epitopes of *T. pallidum* most antigenic protein. Yellow surfaces indicate epitopes, whereas grey sticks represent most of the protein.

Protein folding may break up residues and produce discontinuous or conformational B-cell epitopes. The validated and refined multi-epitope 3D structure was uploaded to the ElliPro server in order to forecast the existence of these epitopes. As demonstrated in Table 7 and Figure 14, this server predicted the presence of seven conformational epitope regions. During vaccine development, the interaction between continuous and discontinuous B-cell epitopes indicated that they are adaptable and therefore can interact with antibodies.

### 3.20. In Silico Immune Simulation

**Figure 15:**
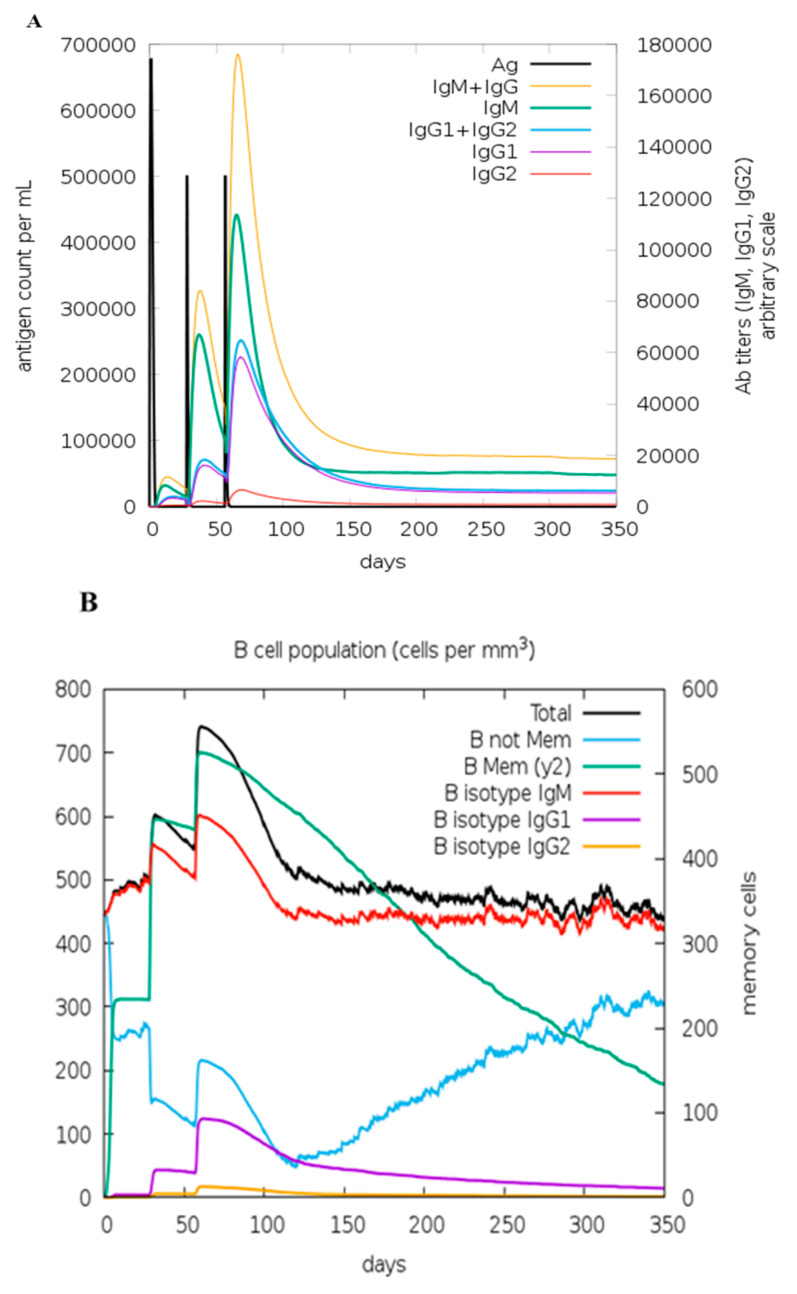

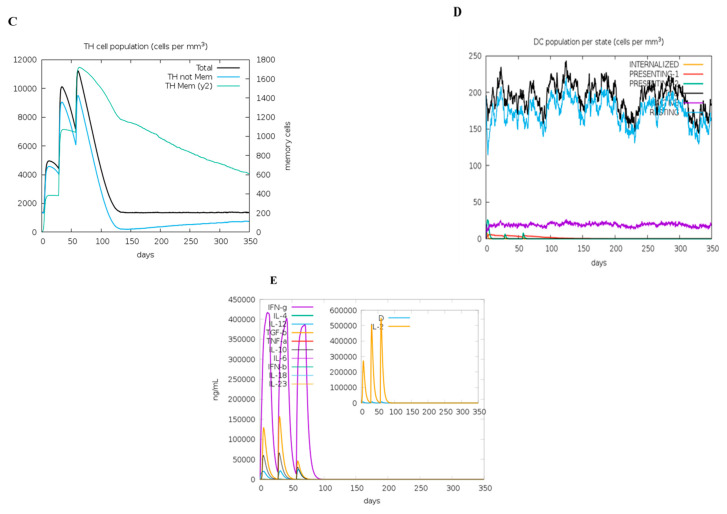
Simulation of the immunological response of the multiepitope-based vaccination construct in silico, (**A**) represents antibody production. In contrast, the black vertical lines show antigen, (**B**) B lymphocytes Population after three injections, (**C**) helper T cell activation throughout the injections, (**D**) Throughout the injections, the active cytotoxic T cell population in each state increased, (**E**) Concentration of cytokines and interleukins with Simpson index D.

The immunological response generated by the C-ImmSim immune simulator against a pathogen was identical to the actual immune response (Figure 15). (Figure 15A) shows that antibody levels (IgM, IgG1, IgG2) levels were more significant in secondary and tertiary reactions, corresponding to fading of antigen concentrations. Long-lasting B cell isotypes were also reported, which demonstrated memory B cell development and swapping ability (Figure 15B). Similarly, memory development was confirmed in the T-helper and cytotoxic T-cell populations, and it was crucial to complement the immune response (Figure 15C). There was a noticeable increase in macrophage activity and interaction and a significant expansion of dendritic cells (Figure 15D). (Figure 15E) shows that there were also elevated levels of interferon-gamma (IFN-γ) and interleukin-2 (IL-2). When cytokine levels increased, the Simpson index D showed high risks, which led to difficulties during the immune response.

### 3.21. Codon Adaptation and In Silico Cloning of the Vaccine Construct

**Figure 16:**
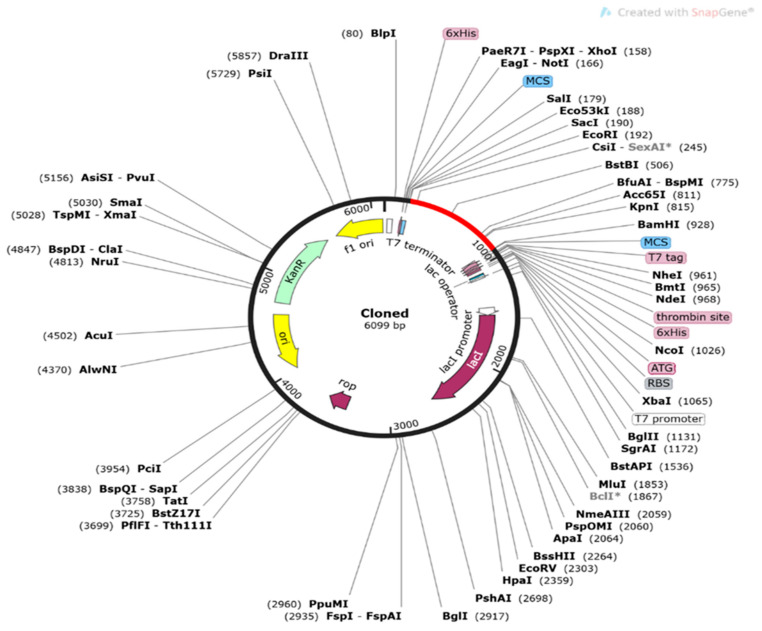
In silico restriction, cloning was used to introduce the final vaccine construct into the pET28a (+) expression vector, in which the red area represents the vaccine insert as well as the black circle represents the vector.

The following vaccination constructions were proposed for the JCat *E. coli* K12 server to facilitate codon adaptation. A codon sequence of 730 nucleotides is ideal. The GC content of the optimal codon sequence was 53.47%. In contrast, the expression levels of the vaccine design in *E. coli* K12 should be between 30 and 70%, and a codon adaption index (CAI) of 0.961 indicates this. The ends of the vaccine gene were modified to include two restriction sites, EcoRI and BamHI. In the end, Snap gene software was used to insert the vaccination gene into the restriction site of the pET28a (+) plasmid (Figure 16). Overall, the clone measured 6099 bp in length.

## 4. Discussion

Sexually transmitted infections (STIs) are caused by various pathogens that are mostly transmitted through sexual intercourse.

Vaccination helps stimulate the immune response as well as defend against pathogen-borne contagious diseases. The prediction and use of surface antigenic epitopes are critical for the development of an efficient vaccine to protect against infectious diseases. Recently, using an in silico technique to work with bacterial pathogens, many targets have been found that are drug resistant or for which no vaccination is available [25]. In the post-genomic age, reverse vaccinology is a common and popular method for quickly identifying new vaccine targets [26,27]. In this study, we mainly used reverse vaccinology and subtractive genomics. We are primarily interested in identifying potential vaccines and therapeutic targets for syphilis disease.

It is worth mentioning that our followed approach has been reported as a successful method for proteome filtration and vaccine candidates selection in several targeted bacteria including *Klebsiella Pneumoniae* [81], *Staphylococcus aureus* [82], *Mycobacterium tuberculosis* [83], *Shigella flexneri* [84], *Pseudomonas aeruginosa* [85] and *Moraxella catarrhalis* [86]. Furthermore, the vaccine generated showed protective functions when it was validated by wet laboratory techniques. For example, a vaccine designed against *Echinococcus granulosus* through an immunoinformatic approach has activated mice humoral immunity and cellular immunity and has good antigenicity and immunogenicity [87]. Furthermore, the evaluation of a multitope vaccine against uropathogenic *Escherichia coli* showed that IgG and IgA antibody levels improved in serum and mucosal samples from vaccinated mice [88]. It is important to mention that the current study has applied an in silico approach for the design and evaluation of the potential vaccine; given the limitations of our method, we were very stringent and only chose top candidate epitopes confirmed by multiple tools. While immunoinformatics integrated with the reverse vaccinology approach was used to propose the potential vaccine of the current study and it was predicted immunogenic, future wet lab experiments are essential to comprehensively validate our findings.

The entire proteome of *T. pallidum* (strain Nichols) was obtained from UniProtKB (UniProt ID: UP000000811) [31]. To prevent an autoimmune response, human homologs were identified and subsequently removed. Furthermore, paralogous, non-essential, non-membrane, and non-virulent proteins were eliminated [33,34]. Antigenic virulence proteins are attractive candidates for the formation of computational vaccines. The ability of a pathogen to infect its host depends on the presence of virulent proteins [42].

Previous research by Zhao et al. 2011, used membrane proteins as vaccine candidates. These researchers found that the outer membrane proteins of *T. pallidum* are, indeed, the main targets of host protective immunity and play a crucial role in the pathogenicity of *T. pallidum* [14,15]. Following the screening, six proteins were identified as promising candidates for vaccine development. It includes the membrane lipoprotein TpN32 (UniProt ID: O07950), DNA translocase FtsK (UniProt ID: O83964), protein Soj homolog (UniProt ID: O83296), site-determining protein (UniProt ID: F7IVD2), ABC transporter, ATP-binding protein (UniProt ID: O83930) and sugar ABC superfamily ATP-binding cassette transporter, ABC protein (UniProt ID: O83782). These proteins were submitted to the STING database to determine their interaction with other proteins [48].

Furthermore, these proteins were subjected to immunoinformatic tools for vaccine design. To choose suitable vaccine candidates, various databases and web servers were used to predict Helper T lymphocytes (HTL), cytotoxic T lymphocytes (CTL), and B cell epitopes. The final epitope sequences for both T- and B-cell cell epitopes were determined. Immunogenicity, toxicity, allergenicity, and antigenicity were all important variables in selecting optimal epitopes.

The vaccine was produced by integrating the CTL, HTL, and B-cell epitopes with the corresponding AAY, GPGPG, and KK linkers. Vaccines need linkers to improve their folding, stability, as well as expression [61]. Multiepitope-based vaccines require adjuvant coupling to boost their immunogenicity, durability, influence stability, immune responses, antigen development, and protection from pathogens. The adjuvant (TLR-2 agonist ESAT6; Accession: AEP68523.1) was linked to the starting location using the EAAAK linker. ESAT6 promotes the TLR2-dependent production of IL-6 and TGF- by dendritic cells. Additionally, it stimulates Th17 immune responses, which are required for optimum vaccination effectiveness. Therefore, the structure of the developed vaccine was subjected to physiochemical assessment. The manufactured vaccine was calculated to have a molecular weight of 48,782.81 kD. The theoretic pI of the construct of 9.50 shows that it is strongly alkaline and provides a steady physiological pH. Furthermore, the aliphatic index and GRAVY score reflect the thermostability and hydrophilicity of the substance. The vaccine has a mean half-life of >10 h in *E. coli* and 10 min in yeast cells (in vivo). Additionally, the vaccination has been described as non-allergenic and highly antigenic. The 2ry and 3ry structures of the protein provide data on its functions, dynamics, and interactions with other proteins or ligands. PSIPRED V3.3 and the Robetta server predict the secondary and tertiary structure of a vaccine. The secondary structure of the developed vaccine consists of 45.22% beta strands, 36.74% Alpha helix, and 18.04% random coil. Numerous validation tools, including ERRAT, ProSA-web, and PROCHECK, were utilized to identify defects in the tertiary structure of the final product vaccine.

The significant interaction between vaccination and innate immune receptors shown by the docking score suggests that the vaccine can activate TLRs. The docking complexes were then subjected to the online server iMODS for molecular dynamics simulation. The MD simulation study of the docked complexes with TLR receptors showed good stability, deformability, and low eigenvalue.

Codon optimization was performed with the Jcat (Java Codon Adaptation Tool) software to enable optimum vaccine expression in the *E. coli* system. We also employed an in silico immune simulator to model vaccine immunological responses in the current study, revealing a good immune response pattern. We administered three doses based on B-cell isotypes and T-cell-mediated immunological reactions, with a significant number of memory B cells with a half-life of several months, whereas repeated vaccination doses improved immune responses. Our simulated immune response indicates a more active immunological response than the first primary dose. IgG and IgM antibody production gradually increases at subsequent and tertiary doses. Numerous vaccinations resulted in prolonged production of IFN- and IL-2, demonstrating that the vaccine effectively induced a response of the humoral immune system to improve immunoglobulin secretion.

Vaccines are formulated using traditional methods, and these vaccines function better in the immune systems of model species. Unfortunately, they are indeed completely ineffective whenever administered to people because of the complexities of the immune system. As a result, using reliable subtractive proteomics and immunoinformatic technologies, this scientific study developed a safe, specific and highly efficient vaccine that could provide long-term protection against Syphilis infections. These vaccines required more clinical trials to verify their vaccine safety and efficacy in vivo.

## 5. Conclusions

*T. pallidum* is among the most common causes of syphilis. This study used immunoinformatics, reverse vaccinology, and subtractive genomics to provide insight into the critical targets of *T. pallidum* for creating a potentially successful vaccine. B- and T-cell epitopes were identified from the pathogenic proteins of *T. pallidum* to develop effective, safe, non-allergenic, extensively antigenic, and specific multiple epitope vaccines. In addition to adjuvant sequences, suitable linkers were applied to improve the stability, effectiveness, and immunological responsiveness of vaccine constructions. The suggested vaccine must exhibit the structural, physicochemical, and immunological qualities required to trigger humoral and cell-mediated immunogenicity. The contact and binding potentials between the receptors (TLR-2 and TLR-4) and the vaccine protein were reported to be stable and high.

Furthermore, simulations of the immune system demonstrated efficient immunogenicity in vivo through reverse translation and codon optimization. To ensure practical expression and durability, the final vaccine was cloned in *E. coli* pET28a + plasmid. The designed vaccine designed requires additional laboratory testing to validate its safety and effectiveness.

## Figures and Tables

**Table 1:** The CD-HIT suite identified paralogous proteins using an 80% threshold.

Cluster	Size	Protein ID	% Similarity
>Cluster 0 0 1			
	215aa 48aa	P56822 O83336	98.14% 93.75%
>Cluster 1 0 >Cluster 2 0			
	756aa 598aa	O83337 O88138	85.45% 81.10%

**Table 2:** Predicted linear cytotoxic T-lymphocyte epitopes, and its major histocompatibility complex class 1 (MHC-I) binding affinity with antigemicity score.

Protein Name	Protein ID	Peptide Sequence	MHC Binding Affinity	Rescale Binding Affinity	C-Terminal Cleavage Affinity	Transport Affinity	Prediction Score	MHC-I Binding	VaxiJen Score	AllerTOP v.2.0	Immunogenicity
FTSK_TREPA DNA translocase FtsK	O83964	LALLGAELY	0.178	0.7558	0.7357	3.047	1.0185	Yes	0.5305	Non-allergen	0.13309
SOJ_TREPA Protein	O83296	TSAINLGAY	0.6054	2.5705	0.4577	2.971	2.7877	Yes	0.4485	Non-allergen	0.18134
TREPA Site-determining protein	F7IVD2	IATNMAIAY	0.2248	0.9546	0.539	3.105	1.1907	Yes	0.6396	Non-allergen	0.0071
TREPA ABC transporter, ATP-binding protein	O83930	TVGFVFQQY	0.1452	0.6164	0.9747	3.011	0.9131	Yes	0.4966	Non-allergen	0.11376

**Table 3:** Predicted helper T-lymphocyte, interferon-gamma (FN-γ) inducing epitopes.

Name	Uniport ID	Start	End	Alleles	Peptide Sequence	Method	Toxicity	Antigenicity	Allergenicity	IFN-γ
FTSK_TREPA DNA translocase FtsK	O83964	26	40	HLA-DRB5*01:01	TLSTFLPLFTLHRAS	Consensus (smm/nn/sturniolo)	Non-toxic	0.589	Non-allergenic	Positive
SOJ_TREPA Protein	O83296	141	155	HLA-DRB4*01:01	VFIPLQCEYFALEGL	Consensus (comb.lib./smm/nn)	Non-toxic	0.7306	Non-allergenic	Positive
TREPA Site-determining protein	F7IVD2	34	48	HLA-DRB1*03:01	KLLLIDPKIVELKLY	Consensus (smm/nn/sturniolo)	Non-toxic	1.3598	Non-allergenic	Positive
TREPA Sugar ABC superfamily ATP-binding cassette transporter	O83782	39	53	HLA-DRB4*01:01	FGLRIRKIPQQEIIR	Consensus (comb.lib./smm/nn)	Non-toxic	0.6532	Non-allergenic	Positive

**Table 4:** Predicted linear B-cell epitopes.

Peptide	Protein	Score	Antigenicity	Conservancy %
PHMQQFNQEHNGDLVSVGNV	TPN32_TREPA membrane lipoprotein TpN32	0.983	0.408	100.00%
GGRVRTYLKERYKGGEVAPA	TPN32_TREPA Membrane lipoprotein TpN32	0.901	0.7478	100.00%
IPAQDDEQGPPRPIPASAAP	FTSK_TREPA DNA translocase FtsK	1	0.6798	100.00%
PSDVHAPASPGSLPSVIPAQ	FTSK_TREPA DNA translocase FtsK	0.998	0.4694	100.00%
TGIKKGPVVTMFELLPPPGI	FTSK_TREPA DNA translocase FtsK	0.996	0.7765	100.00%
PEASAPPEGQFSTEVPLQGG	FTSK_TREPA DNA translocase FtsK	0.99	0.6035	100.00%
RDLMQEKNARERVERHQHRT	TREPA site-determining protein	0.967	0.8618	100.00%
LKDGKIVGDHVRGHGGADGG	TREPA ABC transporter, ATP-binding protein	0.981	1.5311	100.00%
ILGPSGSGKSTCMHMIGCLD	TREPA ABC transporter, ATP-binding protein	0.948	0.9457	100.00%
LQGGTSQVATVHAPPEISTG	TREPA Sugar ABC superfamily ATP-binding cassette transporter	0.966	0.9404	100.00%
RPEAITPRTEETLARECANV	TREPA Sugar ABC superfamily ATP-binding cassette transporter	0.946	0.7421	100.00%

**Table 5:** Protein–protein docking results between TLR-2 and vaccine construct.

Cluster 1	
HADDOCK score Cluster size RMSD from the overall lowest-energy structure Van der Waals energy Electrostatic energy Desolvation energy Restraint’s violation of energy Buried Surface Area Z-Score	−52.2 +/− 6.4 18 2.5 +/− 1.4 −118.9 +/− 12.7 −428.7 +/− 52.0 −4.9 +/− 4.2 1573.1 +/− 143.0 3926.3 +/− 189.9 −2.1

**Table 6:** Protein–protein docking results between TLR-4 and vaccine construct.

Cluster 10	
HADDOCK score Cluster size RMSD from the overall lowest-energy structure Van der Waals energy Electrostatic energy Desolvation energy Restraint’s violation of energy Buried Surface Area Z-Score	17.7 +/− 17.5 5 1.7 +/− 1.6 −40.1 +/− 5.1 −326.1 +/− 93.0 −5.5 +/− 1.8 1286.0 +/− 213.8 2678.3 +/− 408.6 −1.0

**Table 7:** Discontinuous B-cell epitopes predicted by the ElliPro.

No. Residues	Number of Residues Score	Score
1	A:K286, A:S288, A:D289, A:V290, A:H291, A:A292, A:P293, A:A294, A:S295, A:P296, A:G297, A:S298, A:L299, A:P300, A:S301, A:V302, A:I303, A:P304, A:A305, A:Q306, A:K307	0.801
2	A:Q1, A:W2, A:N3, A:F4, A:A5, A:G6, A:I7, A:E8, A:A9, A:A10, A:S11, A:S12, A:A13, A:I14, A:Q15, A:G16, A:T19, A:N63, A:Q66, A:N67, A:L68, A:A69, A:R70, A:T71, A:I72, A:S73, A:E74, A:A75, A:G76, A:Q77, A:A78, A:M79, A:Q80, A:S81, A:T82, A:E83, A:G84, A:N85, A:V86, A:T87, A:G88, A:E89, A:A90, A:A91, A:A92, A:K93, A:L94, A:A95, A:L96, A:L97, A:G98, A:A99, A:E100, A:L101	0.798
3	A:P337, A:E338, A:G339, A:Q340, A:F341, A:V365, A:E366, A:H368, A:Q369, A:H370, A:R371, A:T372, A:K373, A:K374, A:L375, A:K376, A:D377, A:G378, A:K379, A:I380, A:V381, A:G382, A:D383, A:H384, A:V385, A:R386, A:H388, A:G390, A:A391, A:D392, A:G393, A:G394, A:K395, A:K396, A:I397, A:L398, A:G399, A:P400, A:S401, A:G402, A:S403, A:G404, A:K405, A:S406, A:T407, A:C408, A:M409, A:H410, A:M411, A:I412, A:G413, A:C414, A:L415, A:D416, A:K417, A:K418, A:L419, A:Q420, A:G421, A:G422, A:T423, A:S424, A:Q425, A:V426, A:A427, A:T428, A:V429, A:H430, A:A431, A:P432, A:P433, A:E434, A:I435, A:S436, A:T437, A:G438, A:K439, A:R441, A:P442, A:E443, A:A444, A:I445, A:T446, A:P447, A:R448, A:T449, A:E450, A:E451, A:T452, A:L453, A:A454, A:R455, A:E456, A:C457, A:A458, A:N459, A:V460	0.754
4	A:Y129, A:T130, A:V131, A:G132, A:F133, A:V134, A:F135, A:Q136, A:Q137, A:Y138, A:G139, A:P140, A:G141, A:P142, A:G143, A:T144, A:L145, A:S146, A:T147, A:F148, A:L151, A:L154, A:H155, A:A157, A:S158, A:G159, A:P160, A:G161, A:G163, A:Q169	0.64
5	A:T33, A:K34, A:A36, A:A37, A:A38, A:W39, A:G40, A:G41, A:S42, A:G43, A:S44, A:E45, A:Q48, A:Q52	0.612
6	A:S342, A:T343, A:E344, A:V345, A:P346, A:L347, A:Q348, A:K351, A:E358, A:R362	0.57

## Data Availability

The datasets used and/or analyzed during the current study are available from the corresponding author upon reasonable request.
